# Calibrated Automated Thrombinography (CAT), a Tool to Identify Patients at Risk of Bleeding during Anticoagulant Therapy: A Systematic Review

**DOI:** 10.1055/s-0038-1672183

**Published:** 2018-09-26

**Authors:** Suzanne Zwaveling, Saartje Bloemen, Bas de Laat, Hugo ten Cate, Arina ten Cate-Hoek

**Affiliations:** 1 Laboratory for Clinical Thrombosis and Hemostasis, Internal Medicine and Biochemistry, Maastricht University, Maastricht, The Netherlands; 2 Synapse Research Institute, Maastricht, The Netherlands

**Keywords:** thrombin generation, vitamin K antagonist, bleeding, oral anticoagulant

## Abstract

**Background** Bleeding is a feared adverse event during anticoagulant treatment. In patients on vitamin K antagonists, most bleedings occur with the international normalized ratio (INR) in the therapeutic range. Currently, identification of high-risk patients via laboratory methods is not reliable. In this systematic review, we assessed the ability of calibrated automated thrombin generation (CAT-TG) to predict bleeding in patients on anticoagulant treatment.

**Methods** A systematic search was executed in three databases: Medline, Embase, and Cochrane.

**Results** Seven studies were included; two were of good methodological quality. One study showed that patients on warfarin with INRs in range (2–3) admitted for hemorrhage ( *n* = 28), had lower CAT-TG levels (endogenous thrombin potential [ETP]: 333 ± 89 nM/min) than patients on warfarin admitted for other reasons (ETP: 436 ± 207 nM/min; *p* < 0.001). Another study found no difference in ETP or peak levels between bleeding and nonbleeding patients in PPP or PRP. When measured in whole blood, both levels were significantly lower in patients with bleeding compared with nonbleeding patients (median [interquartile range, IQR] ETP: 182.5 [157.2–2,847 nM/min] vs. median [IQR] ETP: 256.2 [194.9–344.2 nM/min]; *p* < 0.001) and median [IQR] peak: 23.9 [19.6–41.8 nM] vs. median [IQR] peak: 39.1 [24.9–53.2 nM]; *p* < 0.05). From the remaining studies, four suggested that CAT-TG is more sensitive in detecting hemostatic abnormalities than INR and one article found ETP and INR to be equally useful. However, insufficient data were provided to validate these conclusions.

**Conclusion** Studies investigating the direct association between decreased CAT-TG values and hemorrhagic events are rare. Therefore, the clinical consequences of low CAT-TG values remain to be further investigated.

## Introduction

Thromboembolic disease is a major cause of morbidity and mortality worldwide. The treatment of thromboembolism relies on anticoagulant drugs of which direct oral anticoagulants (DOACs) and vitamin K antagonists (VKAs) are the most widely prescribed. Although anticoagulant therapy has proven to be effective since many years, it is associated with serious adverse effects. Major bleeding is a feared, but prevalent, complication with an incidence of 1 to 3% annually. 1 Patients on VKA are monitored by measuring the prothrombin time (PT), which is standardized to the international normalized ratio (INR). The INR is used to adjust therapy toward therapeutic levels order to minimize the risk of bleeding. 2 Nevertheless, clinical studies have shown that most bleeding complications occur within a therapeutic INR range. 3 4 5 Rates of major bleedings between 3.09 and 3.4% have been described in patients using warfarin with a time in therapeutic range (TTR) of 66%; for cerebral hemorrhage, these numbers are approximately between 0.38 and 0.7%. 6 7 8 For DOAC, the rates of major bleedings lie between 2.13 and 3.11%, and rates of cerebral hemorrhage are between 0.1 and 0.5%. 6 7 8

Other than INR assessment, which at least helps reduce the risk of bleeding in patients using VKA, no other laboratory test has been helpful in limiting bleeding risk during anticoagulant therapy. The predictive value for bleeding of the PT and the activated partial thromboplastin time (aPTT) has been investigated in several studies, which all found poor correlations between these laboratory tests and hemorrhagic events. 9 10 11 Furthermore, studies have shown that the PT and aPTT are unsuitable to measure the effect of DOAC. In contrast, global coagulation tests, such as calibrated automated thrombin generation (CAT-TG), are able to capture more aspects of the coagulation system, and could potentially be beneficial in assessing the total anticoagulant effects.

By this reasoning, some studies indeed suggested added value of CAT-TG testing in relation to bleeding outcome, 12 or for monitoring of patients on anticoagulant therapy. 13 CAT-TG is able to show the anticoagulant effect of many, possibly all, anticoagulants including DOACs. Multiple studies have previously found that patients who are treated with VKA have a diminished amount of thrombin that is formed over time (low endogenous thrombin potential [ETP]), a lower maximum of thrombin that is formed (low peak height), and a postponed start of coagulation (prolonged lag time) compared with healthy controls. 14 15

We hypothesized that patients who are at risk of bleeding during anticoagulant therapy would have even lower CAT-TG values than patients using anticoagulants who are less at risk of bleeding. In this systematic review, we evaluate whether CAT-TG can detect the risk of bleeding in patients on oral anticoagulant treatment, and as such would be of potential clinical value.

## Methods

### Data Sources and Searches

We performed a systemic search for studies evaluating the use of CAT-TG to predict bleeding in patients on anticoagulant therapy in three databases (Medline, Embase, and Cochrane). The date of the search performed in Medline was May 18, 2017. On May 22, 2017, we systematically searched the Embase and Cochrane databases. No restrictions or filters with regard to language, publication date, and age were applied. The search strategy was refined using keywords of references found in a pilot search and after manual review of reference lists. The keywords included synonyms for “thrombin generation,” “anticoagulant therapy,” and “bleeding” as outcome (see Supplementary Appendix for the complete search strategy). If an article was eligible for full-text reading but could not be retrieved, attempts were made to retrieve the article by searching other libraries or contacting authors. Authors were also contacted in case reports were available only as conference abstracts. The results of the database searches were supplemented by manual review of a reference list of articles that met the inclusion criteria. Duplicate articles were filtered manually.

### Study Selection

**Fig. 1:**
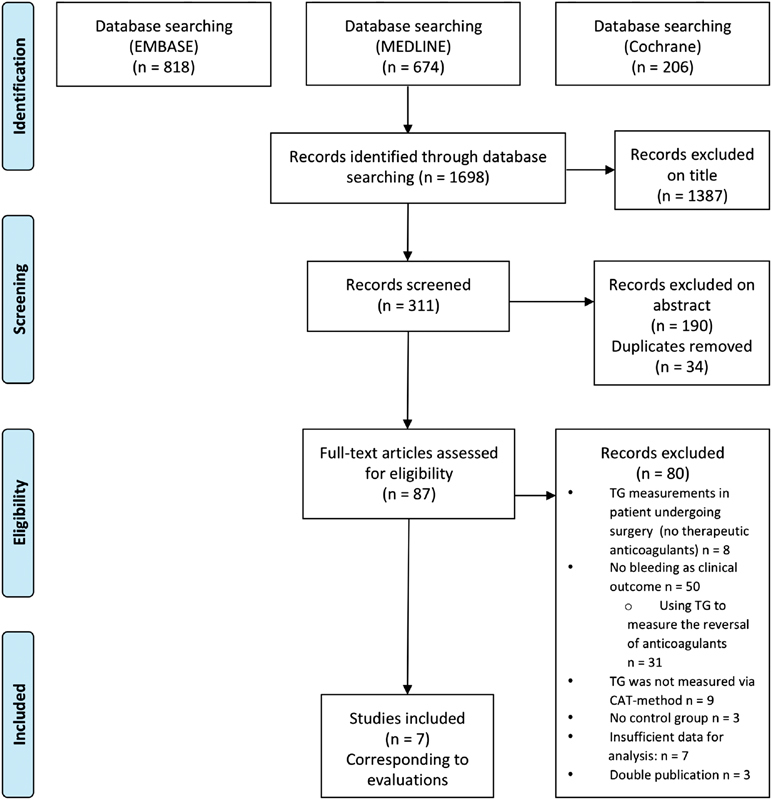
Flow diagram of literature review.

Two reviewers independently screened abstracts between May 22, 2017, until July 2017 and selected articles for eligibility based on predefined inclusion and exclusion criteria. A third reviewer was consulted to agree on the final selection and to resolve any discrepancies between the first two reviewers. For a complete overview of the selection procedures, see Fig. 1 . We included studies that fulfilled the following criteria: (1) research was performed in patients using oral or parenteral anticoagulant treatment for more than 3 months; (2) CAT-TG was measured using a calibrated automated global TG test and the most common parameters: lag time (the time until the first thrombin is formed), peak (the maximum amount of thrombin that is formed), time to peak (time until the maximum is reached), and the ETP (the total amount of thrombin that can be formed over time); (3) there was a clear description of the method of CAT-TG, for example, noting the amount of tissue factor (TF), the use of corn trypsin inhibitor (CTI), thrombomodulin (TM) or activated protein C (APC), the use of phospholipids, and the characteristics of the sample material (platelet-poor plasma [PPP], platelet-rich plasma [PRP], or whole blood); and (4) the outcome of CAT-TG was related to bleeding. Bleedings should have occurred spontaneously, that is, not by a procedure (e.g., postoperative bleeding or a punch biopsy). Additionally, bleeding episodes should have been well documented and described. When a study cohort was described by more than one article, we included only the original data.

Studies were excluded if they (1) consisted of non-original data (e.g., reviews, guidelines, comments); (2) were not written in English; (3) were performed in patients with a known bleeding disorder (e.g., hemophilia), severe liver cirrhosis or liver failure, cancer, or in pediatric patients; (4) were animal studies; (5) did not use the calibrated automated thrombinography (CAT) method as automated global thrombin generation test, but the Technoclone method, measured prothrombin fragment F1 and F2, or thrombin–antithrombin complexes; or (6) did not have “spontaneous clinical bleeding” as the outcome (e.g., postoperative bleeding or punch biopsy).

### Data Extraction and Quality Assessment

The included studies were reviewed in duplicate and data were extracted using a standardized form. The extracted data included author, journal, year of publication, study design, clinical setting, number of patients, patient characteristics including the type of anticoagulant, the method of TG, other types of test used for comparison, bleeding events, follow-up, statistical analysis, and results. It was intended to construct 2 × 2 tables where possible, for the patients with a high/low risk of bleeding, using the extracted numbers of true and false positive as well as negative results according the TG test. Methodological quality of studies was assessed using the Newcastle-Ottawa quality assessment scale for cohort studies and case–control studies. To be able to classify the quality of the articles, we created a table in which we defined the amount of stars. Both are described in the Supplementary Appendix . 16

## Results

### Search Results

The database search yielded 1,698 articles in total: 674 articles were retrieved from Medline, 818 from Embase, and 206 from Cochrane ( Fig. 1 ). A total of 1,387 articles were excluded based on title or abstract. Eighty-seven articles remained eligible and were evaluated based on full text. The majority of studies were not performed in patients on long-term anticoagulant treatment and/or did not assess the direct relation between CAT-TG and clinical bleeding in the absence of an intervention and were therefore excluded ( *n* = 50). For example, 31 of these studies assessed TG as a tool to investigate the reversal of anticoagulation. The examined drugs in these studies were often DOACs and tests were mostly performed in healthy subjects. These studies used the normalization of coagulation tests (such as CAT-TG) as an indication that coagulation was restored, but did not investigate the relation with clinical bleeding. 13 17 18 19 20 21 22 23 24 25 26 27 28 29 30 31 32 33 34 35 36 37 38 39 40 41 42 43 44 45 Eight studies investigated the role of TG in prediction of bleeding after cardiac surgery. 46 47 48 49 50 51 52 53 These studies were excluded because all patients underwent surgical intervention; therefore, the outcome was not compatible with spontaneous hemorrhage ( *n* = 8). We did not find any studies investigating DOACs and the direct relation between TG and clinical bleeding. Finally, seven articles were included. All articles studied the value of CAT-TG to evaluate a bleeding risk in patients using VKAs and two of these are currently unpublished. We approached the authors, but unfortunately no additional information could be retrieved.

### General Aspects and Results of the Included Studies

The studies selected were mostly prospective studies. There are some similarities between the studies concerning the choice of anticoagulants, the target range for the INR, and the investigated method of TG. There was heterogeneity in patient selection, duration of follow-up, and also in the registration of outcome parameters. A general overview of the included studies is given in Table 1 . More specific data according CAT-TG values are displayed in Table 2 . The quality of the included studies was rated according to the Newcastle-Ottawa Quality assessment scale ( Tables 3 and 4 ).

**Table 1:** Summary of included studies

Study	Design	Population Age (median + range)/(mean + SD)	Type of AC INR range	CAT method	Outcome	Results	Conclusion
Bloemen et al 2017	Prospective cohort study	129 patients 70 y [62–76]	Acenocoumarol (95%) Phenprocoumon (5%) 103 within range: 2–3 26 within range: 3–4 Total TTR 79.9%	PPP/PRP 1 pM TF 5 pM TF (±TM) WB 1 pM TF	Bleeding	26/129 patients suffered from bleeding - ETP or peak levels in PPP or PRP CAT could not discriminate between bleeding and nonbleeding patients - WB showed that patients who suffered from bleeding ( *n* = 26) had significantly lower ETP ( *p* < 0.01) and peak ( *p* < 0.05) (median: 182.5 nM/min and 23.9 nM, respectively) compared with patients who did not bleed ( *n* = 203) (median: 256.2 nM/min and 39.1 nM)	INR and plasma CAT-TG did not discriminate between bleeding and nonbleeding patients CAT-TG measured in WB is able to detect patients at risk of bleeding when treated with VKA
Luna-Záizar et al 2015	Retrospective cohort study	43 patients 41.1 y (±11.3)	27 patients using VKA (warfarin or acenocoumarol) INR range: 2.0–3.0 1 using LMWH 5 using antiplatelet therapy (aspirin or clopidogrel) 10 without	PPP 5 pM TF	Bleeding, thrombosis	1/27 patients died from uncontrolled hemorrhage, showing an INR of 3.47 while the ETP was 11.9% of normal (100%) and the peak 11.4%	CAT-TG showed excellent sensitivity and specificity to assess the anticoagulant status in primary thrombophilia patients treated with VKA, and those without anticoagulant or other treatment
Marchetti et al 2013	Prospective cohort study	90 patients 73 y (±9)	Warfarin 77 within range: 2–3 13 within range: 3–4	PPP 5 pM TF (±APC)	Bleeding, Thrombosis APC resistance	2/90 patients suffered from bleeding during follow up (2 years) while in range; 1/2 bleeding cases: INR 2.54, ETP 184 nM/min	Wide CAT-TG variability in subjects with similar INR values, suggest higher sensitivity of CAT-TG in detecting hemostatic abnormalities
Choi et al 2013	Retrospective cohort study	239 patients 61 y [26–89] (mean)	Warfarin for at least 6 mo INR range: 2.0–3.0	PPP 1–5 pM TF	Bleeding, thrombosis	- 38/239 patients experienced minor bleedings. Unknown is if their ETP was lower than patients without bleeding. - In the adequate anticoagulation range, minor bleeding rates were 6.5 and 5.4% using the INR and ETP, respectively	INR and ETP both show similar efficacy for warfarin monitoring with respect to the clinical complication rate
Verzeroli et al 2014	Prospective cohort study	40 patients age: not described	20 using warfarin 13 within range: 2–3 7 within range: 3–4 All 100% TTR during last 3 mo 20 healthy controls	PPP 10 pM TF	Bleeding, APC resistance	4/20 patients suffered from bleeding 1/4 = major bleeding, INR was within therapeutic range, but ETP levels were very low	The variability of CAT-TG values in patients with similar INR and the occurrence of bleeding complications in patients in therapeutic PT-INR suggests that CAT-TG can be more sensitive in detecting a hemorrhagic phenotype
Herpers et al 2015	Retrospective open, observational, case–control study	126 patients 86 patients using VKA, and eligible for reversal 40 healthy controls 83 y [56–99]	Phenprocoumon range: not described	PPP 5 pM TF	Bleeding	57/86 patients presenting with bleeding, had a significant lower median ETP (230 nM/min, [0–826]) than 29/86 patients in need of VKA reversal because of upcoming surgery, without bleeding (median ETP 321 nM/min, [0–663], *p* = 0.03) There was no significant difference in lag time of peak between these groups	CAT-TG-based calculations may enable a more accurate PCC dosing regimen for patients using VKA compared with INR ETP is not significantly different between patients experiencing bleeding and patients requiring surgery
Dargaud et al 2013	Prospective cross-sectional study	341 patients 28 admitted for bleeding 74 y (±14) 13 admitted for thrombosis 73 y (±19) 300 admitted for other reasons (control) 76 y (±13)	Warfarin INR range: 2.0–3.0	PPP 1 pM TF	Bleeding Thrombosis	28/341 patients on warfarin who were admitted for hemorrhage, had significantly lower CAT-TG levels (ETP 333 ± 89 nM/min) than 300/341 control patients on warfarin who were admitted for any other medical condition (ETP 436 ± 207 nM/min) ( *p* < 0.001), despite similar INR values (2–3)	CAT-TG is thought to be more suitable to assess global hemostatic adequacy than common clinical coagulation tests (PT and aPTT) and probably provide a more sensitive measure of the level of anticoagulation

Abbreviations: AC, anticoagulants; APC, activated protein C; aPTT, activated partial thromboplastin time; CAT-TG, calibrated automated thrombin generation; ETP, endogenous thrombin potential; INR, international normalized ratio; LMWH, low-molecular-weight heparin; PPP, platelet-poor plasma; PRP, platelet-rich plasma; PT, prothrombin time; SD, standard deviation; TF, tissue factor; TM, thrombomodulin; TTR, time in therapeutic range; VKA, vitamin K antagonists; WB, whole blood.

**Table 2:** CAT-TG values compared in bleeding and nonbleeding patients using VKA

			Bleeding				Nonbleeding		
Author	CAT-TG	N		Median/Mean	IQR/SD	N	Median/Mean	IQR/SD	*p* -value
Bloemen et al	WB	26	ETP (nM/min)	182.5	157–285	101	256.2	195–344	<0.01 a
			Peak (nM)	23.9	20–42		39.1	25–53	<0.05 b
			TtPeak (min)	12.2	10–18		12.2	10–15	0.427
			Lag time (min)	7.1	5–8		6.5	5–8	0.545
	PPP	26	ETP (nM/min)	367	298–501	101	438	344–541	0.09
			Peak (nM)	76	59–99		86.6	63–114	0.181
			TtPeak (min)	7.8	6–10		8.3	7–10	0.897
			Lag time (min)	5.1	4–7		5.5	4–7	0.805
Luna-Záizar et al	PPP	1	ETP % of normal (normal = 100%)	**11.9%**		26	**31.2%**	**±12**	n/a
			Peak % of normal) (normal = 100%)	**11.4%**			**32%**	**±10**	n/a
			Lag time (min)	n/a			**6**	**±2**	n/a
Marchetti et al	PPP	2	ETP (nM/min)	** 184 c**		90	**435**	**±190**	n/a
			Peak (nM)	**76**			**109**	**±51**	n/a
			TtPeak (min)	n/a			**6**	**±2**	n/a
			Lag time (min)	n/a			**4**	**±2**	n/a
Choi et al	PPP	38		n/a		239	n/a		n/a
Verzeroli et al	PPP	4		n/a		20	n/a		n/a
Herpers et al	PPP	57	ETP (nM/min)	230	(0–826)	29	321	(0–663)	0.03 b
			Peak (nM)	27	(0–208)		42	(0–145)	>0.05
			Lag time (min)	12	(3 to >60)		8	(4 to >60)	>0.05
Dargaud et al	PPP	28	ETP (nM/min)	**333**	**±89**	300	**436**	**±207**	<0.001 a

Abbreviations: CAT-TG, calibrated automated thrombin generation; ETP, endogenous thrombin potential; IQR, interquartile range; n/a, not applicable; N, number; PPP, platelet-poor plasma; SD, standard deviation; TtPeak, time to peak; WB, whole blood; VKA, vitamin K antagonist.

Note: Mean values with SD are in bold; the other values are medians with interquartile ranges.

a Significant difference, *p* < 0.01.

b Significant difference, *p* < 0.05.

c Only CAT-TG values from one patient were reported, although two patients suffered from bleeding.

**Table 3:** Newcastle-Ottawa Quality Assessment Scale—cohort studies

	Representativeness exposed cohort	Selection of nonexposed cohort	Ascertainment of exposure	Outcome of interest not present at the start of study	Overall score for selection	Comparability of cohort on the basis of design or analysis	Overall score for comparability	Assessment of outcome	Follow-up long enough for outcomes to occur	Adequacy of follow-up cohorts	Overall score for outcome	Total score	Percentage
Bloemen et al 2017	★	★	★	−	3	★★	2	★	★	−	2	7/9	77.7
Luna-Záizar et al 2015	★	★	★	−	3	−	0	★	−	−	1	4/9	44.4
Verzeroli et al 2014	−	★	★	−	2	★	1	−	★	−	1	4/9	44.4
Choi et al 2013	★	★	★	−	3	★	1	★	−	−	1	5/9	55.5
Marchetti et al 2013	−	★	★	−	2	★	1	−	★	−	1	4/9	44.4

**Table 4:** Newcastle Ottawa Quality Assessment Scale—case–control studies

	Case definition adequate?	Representativeness of the cases	Selection of controls	Definition of controls	Selection	Comparability of cases and controls on the basis of design or analysis	Comparability	Assessment of exposure	Same method of ascertainment for cases and controls	Nonresponse rate	Exposure	Total score	Percentage
Herpers et al 2015	★	−	★	−	2	−	0	★	★	n/a	2	4/8	50
Dargaud et al 2013	★	★	★	−	3	★	1	★	★	n/a	2	6/8	75

Abbreviation: n/a, not applicable.

#### Cohort Studies

Bloemen et al studied whether CAT-TG either in plasma or in whole blood could be used to predict bleeding episodes in patients using VKA. 54 The authors included 129 patients who used VKA for at least 3 months with an average age of 68 years. Of this population, 21.7% were female and 95.4% of the patients were treated with acenocoumarol. The average TTR was 79.9%. Of the included patients, 103 were classified in the lower INR range (2.0–3.0) and 26 patients in the higher range (2.5–4.0). Patient characteristics were well defined and there was a follow-up of 15.5 months. As main outcome, clinically relevant bleeding episodes were scored during follow-up according to the criteria defined by the Dutch Federation of Anticoagulation Clinics (FNT). Clinically relevant bleeding episodes occurred in 26 (20.2%) patients. A total of 44 bleeding episodes were registered, which is due to the fact that some patients had multiple episodes. CAT-TG measured in PPP with 5 pM TF showed lower ETP levels in patients who had experienced bleeding compared with patients without bleeding; however, this difference was not significant. Also other CAT-TG parameters like peak height could not discriminate between bleeding and nonbleeding patients ( *p* = 0.18) nor could INR ( *p* = 0.87), hematocrit ( *p* = 0.23), hemoglobin ( *p* = 0.11), or fibrinogen level ( *p* = 0.54). In contrast, an increased HAS-BLED score was significantly associated with bleeding ( *p* <0.05). Interestingly, CAT-TG stimulated with 1 pM TF in whole blood yielded significantly lower ETP ( *p* < 0.01) and peak height levels ( *p* < 0.05) in patients that suffered from bleeding, compared with patients who did not bleed.

Luna-Záizar et al explored the usefulness of CAT-TG to assess the anticoagulation status compared with the INR in patients with primary thrombophilia. 55 Fifty patients, who were diagnosed with inherited thrombophilia and had experienced at least one thrombotic event, were included. Whether the selection was consecutive is not described, neither are the criteria for exclusion. Definitions of the outcome “bleeding” (major or minor) were not further explained. Complete results were obtained from 43 patients (13 males [30%]/30 females [70%], mean age: 41.1 ± 11.3 years). Twenty-seven of these patients were treated with VKA and 1 patient was treated with low-molecular-weight heparin (LMWH). Fifteen patients were either without anticoagulation medication or used antiplatelet therapy only. CAT-TG was initiated with 5 pM TF in PPP. During the study, only one bleeding event occurred. This patient on VKA treatment suffered from fatal hemorrhage. While the INR was 3.47, ETP and peak levels were lower than CAT-TG values in patients using VKA (2.0–3.0) who did not bleed. Furthermore, the study showed significant lower ETP and peak values for patients who used VKA or LMWH compared with patients who were untreated ( *p* < 0.0001). Lag time was also discriminative between these groups but displayed great interindividual variability in the group of patients under optimal anticoagulation with VKA (INR: 2.0–3.0). An inverse nonlinear relation was found between ETP values ( *R* ^2^ = 0.649), peak ( *R* ^2^ = 0.633), and the velocity index ( *R* ^2^ = 0.532) versus INR values in patients treated with VKA. As expected, a positive linear correlation between lag time and INR was found ( *R* ^2^ = 0.338).

Marchetti et al performed a prospective cohort study to investigate the characteristics of CAT-TG in patients with atrial fibrillation (AF) on permanent oral anticoagulation therapy with warfarin. 56 They included 90 patients (56 males/34 females; aged 73 ± 9 years) of whom 77 patients had a target INR within 2.0 to 3.0 range, and 13 patients within 3.0 to 4.0 range. Other patient characteristics such as concomitant use of antiplatelet therapy were not presented. Outcomes of interest were bleeding and thrombosis, but predefined criteria for bleeding (minor or major) or thrombosis were not described in detail. Patients were followed up for an average time of 2 years. During follow-up, two bleedings occurred: one patient suffered a gastric bleeding and another patient experienced cerebral hemorrhage, both of them being within the INR target range. One of these patients with an INR of 2.54 did have a low ETP value. CAT-TG was performed using 5 pM TF. Besides the ETP, other parameters were also evaluated: peak height, lag time, and time to peak. A significantly decreased ETP (435 ± 190 vs. 1,229 ± 114 nM/min) and peak (109 ± 51 vs. 256 ± 44 nM) were observed in patients with stable INRs in their appropriate range, compared with healthy controls ( *p* < 0.01). In addition, lag time and time to peak were significantly prolonged in the patient group (4.1 ± 1.5 vs. 2.0 ± 0.3 minutes and 6.2 ± 1.7 vs. 4.6 ± 0.6 minutes, respectively).

A moderate correlation between INR and all CAT-TG parameters ( *R* ^2^ = 0.6) was found, but patients with similar INRs showed a large variability in TG levels, particularly the patients within target INR range of 2.0 to 3.0.

Choi et al compared the monitoring performance of ETP with INR. 57 In this retrospective cohort study, 239 patients (129 males/110 females; mean age: 61 years, range: 26–89 years) were included using warfarin for at least 6 weeks for several indications (prosthetic heart valves, AF, ischemic heart failure, or DVT). The INR ranged from 1.0 to 4.26. Exclusion criteria were younger than 19 years, the use of other anticoagulant treatment than warfarin, or the use of antiplatelet agents. Outcome was bleeding and thrombosis, obtained from medical records. TG was performed using the CAT method. The amount of TF and PL was not clearly mentioned, but a reference was made to the article of Hemker et al in which 5 pM TF and 4 μM PL were used. 58 A significant inverse correlation between ETP and INR was established ( *r* = −0.769, *p* < 0.001). With a regression equation, the authors calculated a therapeutic range for the ETP (290.1–494.6 nM/min), which would correspond to the INR range from 2.0 to 3.0. Subsequently, the patients were divided into three groups (under-, adequately, and over-anticoagulated) according to their anticoagulation status judged by the INR or ETP. Whether the ETP is measured per individual or calculated based on the formula mentioned earlier remains uncertain. To assess the monitoring performance of the INR and ETP, they compared their anticoagulation status to clinical complications rates. When the division to the adequate anticoagulation group was based on INR or ETP, bleeding rates were 6.3 and 5.4%, respectively. Although the use of ETP as target parameter yielded a lower bleeding rate, the difference was not statistically significant. During the study period, only minor bleedings occurred in 38 patients. Individual ETP values were not reported in the article.

Verzeroli et al investigated the correlation between CAT-TG and INR in patients treated with warfarin and whether CAT-TG could be useful to identify subjects at a higher risk of bleeding. 59 The study included 20 patients using warfarin (13 for AF, 7 for cardiac valve prosthesis) who had been in their appropriate target INR ranges for 100% over the last 3 months; 20 healthy subjects were studied as controls. Patient characteristics were not described, nor were INR target ranges specified. Patients were followed up for 1 year. CAT-TG when activated with 10 pM TF, showed a significantly lower TG potential in the patients using warfarin compared with healthy controls; a significant correlation between the INR levels and all TG parameters ( *p* < 0.01) was mentioned, but the actual data on correlations were not given. During follow-up, bleeding complications were registered in four patients, of which one was major, while all patients were in their respective target INR ranges. All four patients had a very low TG potential, but exact levels of CAT-TG parameters are not described. The diagnostic criteria of “bleeding” were not further defined.

#### Case–Control Studies

Herpers et al compared the use of the INR with CAT-TG to guide VKA reversal. 35 In an open, observational study, they studied 86 patients treated with phenprocoumon, who were in need of VKA reversal. The median age was 83 years (range: 56–99 years), 53% were female and the indication for VKA was mostly cardiac arrhythmia (84%). They compared 29 patients who needed prophylactic VKA reversal because of upcoming surgery with 57 patients who needed VKA reversal because of hemorrhage. Coagulation reactions during CAT-TG were initiated with 5 pM TF. A significantly lower ETP was described in bleeding patients compared with nonbleeding patients. Lag time and peak did not differ significantly ( *p* >0.05) between these groups. However, in the Discussion and Conclusion sections, the authors stated that no significant difference was found in any CAT parameter (including the ETP).

Dargaud et al evaluated the hypothesis that the INR might underestimate the level of anticoagulation in patients with a lower factor IX level in contrast to CAT-TG, suggesting CAT-TG to be a more accurate test. 60 In this study, 341 patients on warfarin with a stable INR between 2.0 and 3.0 were included at admission to the emergency department. Twenty-eight of these patients (18 males/10 females, aged 74 ± 14 years) were admitted for hemorrhage. Thirteen patients were admitted for thrombosis and were not taken into account in this review. Three hundred patients were admitted for other medical reasons (151 males/149 females, aged 76 ± 13 years). Of the 28 patients who were admitted for hemorrhage, 22 had major bleeding (7 spontaneous intracranial hemorrhage, 3 trauma-related intracranial hemorrhage, 12 with gastrointestinal tract bleeding); 5 patients exhibited muscle hematomas, severe epistaxis, or gum bleeding; and 1 patient had urinary tract bleeding. All patients were reviewed for potential underlying disorders that might explain their bleeding episodes, but no significant abnormalities were detected. The criteria for major bleeding were not explained in further detail. All CAT-TG experiments were activated with 1 pM TF. Patients on warfarin who were admitted for hemorrhage ( *n* = 28) had significantly lower TG levels than patients on warfarin who were admitted for any other medical condition, while having similar INRs in target range. No significant correlation between the INR levels in the range of 2.0 to 3.0, and ETP values ( *r* = −0.05, 95% CI: = −0.168 to 0.059; *p* = 0.361) was found. Mean TG levels of all admitted patients on warfarin with INRs in the target range of 2 to 3 ( *n* = 341) were significantly lower than TG levels of 100 healthy controls (ETP 428 ± 200 nM/min vs. ETP 1,380 ± 214 nM/min).

### Thrombin Generation Methods in the Included Studies

All studies measured TG by using the CAT method, although this method was not always adequately described. Bloemen et al, 54 Luna-Záizar et al, 55 Choi et al, 57 Herpers et al, 35 and Dargaud et al 60 all used the CAT method propounded by Hemker et al. 58 The assays were performed on a Fluorscan Ascent fluorometer (Thermo Labsystems OY, Helsinki, Finland). The software program, Thrombinoscope (Thrombinoscope BV), enabled the calculation of thrombin activity. Bloemen et al used 1 pM as well as 5 pM TF to initiate coagulation in PPP. Dargaud et al used 1 pM TF. 60 Both Luna-Záizar et al 55 and Herpers et al 35 used 5 pM TF, while Choi et al 57 did not mention the used amount of TF. Bloemen et al measured CAT-TG also in whole blood, using 1 pM TF, according the specifications of Ninivaggi et al. 61 Marchetti et al and Verzeroli et al did not describe the CAT method in detail. They did report the amount of TF used, which was 5 and 10 pM , respectively.

All studies used the same concentration of phospholipids (4 μM). Two studies (Bloemen et al 54 and Dargaud et al 60 ) reported the specifications of the type of phospholipids used; both used phospholipids obtained from Avanti Polar Lipids (Alabaster, AL, United States).

## Discussion

Hemorrhagic complications remain the most important concern during the management of anticoagulation therapy. Insight into the (individual) bleeding risk of patients would likely affect clinical decision making. However, sensitive instruments to identify patients at increased risk of bleeding, who would benefit from more careful management, are lacking. CAT-TG measures the total amount of thrombin formed over time, and offers a more global assessment of coagulation because it is influenced by all clotting factors involved in the cascade. 62 Several studies have investigated the role of TG as a new tool to assess coagulation. 13 15 63 64 This review was performed to assess the currently available evidence for the predictive ability of CAT-TG in relation to bleeding, not related to surgery, in patients on anticoagulant treatment.

We retrieved 1,698 articles when searching the key words “thrombin generation,” “anticoagulant therapy,” and “bleeding.” Interestingly, most studies correlated CAT-TG to the INR, based on the known association between INR and risk of bleeding, to indirectly link bleeding risk to CAT-TG values. In our search, we also identified 31 articles exploring the value of TG for the assessment of the reversal of different anticoagulants, including the DOAC drugs. TG levels were used as reference point to determine whether coagulation was restored after adding three- or four-factor prothrombin complex concentrate (PCC), fresh frozen plasma (FFP), or factor VIII inhibitor bypass activity (FEIBA). 13 17 18 19 20 21 22 23 24 25 26 27 28 29 30 31 32 33 34 35 36 37 38 39 40 41 42

Remarkably few studies investigated the direct relation between CAT-TG values and clinical bleeding in stable anticoagulated patients, and could therefore be included in this systematic review. Of these seven studies, only two studies were of good methodological quality. The quality of the other studies was considered as moderate. The data available from the studies were unfortunately insufficient to perform a meta-analysis. There are multiple techniques to measure TG. In this review, we chose to solely evaluate TG measured by the CAT method. The difference between methods, as well in protocol as in reagents, makes the TG data between techniques hard to compare. Since TG measured with CAT was previously found to be correlated to a hypocoagulable, as well as a hypercoagulable state in patients, we decided to investigate TG measures with CAT only, although this would limit our scope. 51 65 66 Moreover, to fairly compare outcomes between different studies, a standardized protocol of the CAT method is important. Although the protocols of the included study were largely identical, two studies used different amounts of TF in CAT, which made comparison between outcomes of studies difficult. The use of lower amounts of TF (1 pM) will enhance the sensitivity of the assay, but could reduce the reproducibility. Choosing a high concentration of TF (e.g., 10 pM ) , the contribution of the intrinsic pathway to coagulation will become negligible. This review stressed the need for an unambiguous study protocol for CAT-TG.

Some of the included studies were limited because of a small sample size. Additionally, in all included cohort studies, the number of patients who suffered from bleeding during the follow-up was lower compared with the patients who did not bleed. Consequently, the number of bleeding events may have been too low to reach solid conclusions about the predictive value of CAT-TG for bleeding.

The study by Dargaud et al found significantly lower mean ETP levels in patients experiencing warfarin-related hemorrhage compared with patients with similar INR values in the desired range, who did not experience bleeding. This study had sufficient power and enough clinical bleedings (8.2%) to point out significant differences in ETP levels between bleeding and nonbleeding patients. However, the design of this study may not be suitable to answer the question whether low CAT-TG values are predictive for bleeding. In this cross-sectional study, the patients suffered from warfarin-related hemorrhage at the time of inclusion and blood withdrawal for the study assessment. Therefore, the ETP levels most likely represent a more acute hemostatic condition, involving bleeding-associated consumption of coagulation proteins.

Herpers et al investigated whether patients in need of VKA reversal because of bleeding had lower ETP than patients in need of VKA reversal who did not bleed. 35 This study was considered of moderate methodological quality, even more so because in the discussion and the conclusions of the article, contradictory interpretations of the findings were presented. In addition, the difference in design makes it difficult to compare the results of previous studies with the other included studies, which were cohort studies. In the cohort studies, the ETP levels are measured at inclusion while patients are in a stable, nonbleeding state, and bleedings are registered during follow-up. The latter design is more informative on the predicting ability of CAT-TG. Unfortunately, out of the five included prospective cohort studies, only two studies were of acceptable methodological quality. Both the studies of Marchetti et al and Vezeroli et al suggested that the differences in ETP values found in patients with similar INRs indicate that CAT-TG is more sensitive in detecting hemostatic abnormalities than INR. However, data were derived from conference abstracts only; therefore, information was incomplete. Luna-Záizar et al examined the use of CAT-TG in patients using VKA to assess the anticoagulation status compared with the INR, which corresponds to our research question. 55 The quality of the study was rated as moderate. The sample size was small and only one (major) bleeding event was described during follow-up. This patient had lower ETP values compared with other patients with similar INRs but did not bleed. The authors state that this clinical event suggests that the ETP can predict an increased hemorrhagic risk, making CAT-TG a better monitoring parameter than the INR. However, these conclusions are based on a single event rendering the outcome highly questionable. The research question of Choi et al was also in accordance with ours. 57 The sample size of this study was sufficient and patients as well as controls were drawn out of the community. At the same time, the follow-up was poorly described. Moreover, the individual ETP values of patients could not be extracted from the data, making it impossible to compare ETP between bleeding and nonbleeding VKA patients. Patients were divided into groups based on their INR or ETP values. The calculation made to develop a therapeutic range for ETP, which should compare with the INR range 2.0 to 3.0, was partly based on the INR. Although the article shows an inverse correlation between INR and ETP, which is linear between the INR range 2.0 and 3.0, it cannot be assumed that this correlation will remain linear over a larger range. 15 67 Therefore, ETP values comparable with INRs outside the range 2.0 to 3.0 cannot be justified. Overall, the results of this study were not suitable to answer our research question mainly because of the design despite the general moderate quality. The study by Bloemen et al was considered of fairly good methodological quality. The design was suitable to answer our research question and gave a detailed description of inclusion criteria, patient characteristics, and the specific execution of the CAT-TG test. Although lower average ETP levels were seen in the patients who experienced bleeding compared with patients who did not, no significant difference was observed in the PPP CAT-TG. It could be argued that this study was underpowered, and therefore not able to answer the hypothesis if CAT-TG can predict bleeding in patients. When a larger number of patients would have been investigated, a significant difference might be found, but a substantial overlap in ETP values between groups will remain, rendering CAT-TG in PPP probably not discriminative between bleeding and nonbleeding patients. One of the limitations of this study was a lack of correction for several confounding factors that are associated with a higher bleeding risk, for example, diabetes mellitus, a reduced kidney function, or a positive bleeding history. Additionally, the occurrence of bleeding complications during follow-up was higher than expected (20.2%) and most bleedings were clinically relevant minor bleeds. Previous studies have shown that minor bleedings during anticoagulant therapy can be predictive for subsequent major bleedings, although the underlying causal mechanism for this is still to be elucidated. 68 69 Some evidence indicates that minor bleedings are a marker for fixed and currently unknown risk factors for major bleeding events. In the study by Bloemen et al it was shown that CAT-TG measured in whole blood was able to distinguish patients at risk of bleeding. One explanation for these findings could be the concurrent effect of platelets and red cells in the whole blood thrombin generation assay. Since a previous study from our laboratory did not detect any significant influence of platelets on bleeding risk in anticoagulated patients, by demonstrating in PRP that platelet function tests and von Willebrand factor levels did not differ between bleeding and nonbleeding patients on VKA 70 a contribution from red blood cells or leukocytes is likely.

Although the findings of Bloemen et al in whole blood are promising, whole blood TG is only recently developed, and the present study provides the first implementation in patients on VKA. More studies are needed to see if these results can be confirmed.

To genuinely evaluate the predictive ability of CAT-TG in relation to bleeding events, it is required to investigate the sensitivity, specificity, and positive and negative predictive value of the assay, to calculate an odds ratio or construct a receiver operation characteristic (ROC) curve. Unfortunately, this was not possible due to the absence of predefined cutoff values of CAT-TG parameters and because most articles compared mean or median CAT-TG values between patients who suffered from bleeding and patients who did not. Individual CAT-TG values of bleeding and nonbleeding patients were not reported.

Unfortunately, no articles were found investigating the value of CAT-TG to predict bleeding in patients using the DOACs. This could be explained by different reasons. First, measuring direct IIa inhibitors with TG shows a paradoxical increase of the peak and ETP and is therefore currently not reliable enough. 71 72 Another explanation could be that measurements of direct Xa inhibitors, although possible with CAT-TG, have not yet been correlated to clinical bleeding complications in humans.

## Conclusion

CAT-TG is frequently used to assess the effects of anticoagulation in different research settings. Clinical studies are mostly performed in patients treated with VKA, showing decreased TG values in patients compared with healthy controls. 14 58 60 73

The studies of Bloemen et al and Dargaud et al, which were found to be of the highest methodological quality according this review, both found evidence supporting an association between low CAT-TG values and bleeding in patients using VKA. Other reviewed studies agreed with this reasoning, but did not provide enough data to validate this hypothesis. Unfortunately, studies investigating the direct association between decreased CAT-TG values and actual hemorrhagic events are scarce; therefore, the clinical consequences of low CAT-TG values remain to be investigated. To further evaluate whether low CAT-TG values can identify patients with a higher bleeding risk, new studies are needed.

While application in VKA treatment is an interesting avenue to pursue, it may be even more important to look for associations between CAT-TG activity and bleeding outcomes in patients using DOACs. The use of fixed doses of DOAC, based solely on patient characteristics, causes a wide variability in their anticoagulant responses, which makes testing with overall assays like CAT-TG potentially interesting.
