# Supplementary material for: Calibrated Automated Thrombinography (CAT), a Tool to Identify Patients at Risk of Bleeding during Anticoagulant Therapy: A Systematic Review
Source: TH Open. 2018 Sep 26;2(3):e291–302. doi: 10.1055/s-0038-1672183 (PMC6524885; doi:10.1055/s-0038-1672183)
Supplement: Supplementary file 1 — Supplementary Material [file 10-1055-s-0038-1672183-s180018.pdf]

Supplementary Appendix

Search

The search was performed using the following terms:

((((((((((((((((((((("anticoagulated patients") OR "Anticoagulant therapy") OR "Anticoagulant treatment") OR "Antithrombotic therapy") OR "Anticoagulants") OR "Anticoagulant drugs") OR "Anticoagulant agents") OR "Antithrombotic agents") OR Warfarin) OR Anticoagulants) OR (("Anticoagulants/administration and dosage"[Mesh] OR "Anticoagulants/therapeutic use"[Mesh] OR "Anticoagulants/therapy"[-Mesh]))) OR "Warfarin/therapeutic use"[Mesh]) OR "Vitamin K antagonists") OR "Oral vitamin K antagonist therapy") OR ("Vitamin K/antagonists and inhibitors"[Mesh])) OR "International normalized ratio") OR ("Oral anticoagulants" OR "Direct oral anticoagulants" OR "Non vitamin K oral anticoagulants")) OR ("Unfractionated heparin" OR heparin)) OR ("Low molecular weight heparin" OR LMWH)) OR fondaparinux)) AND (((((((("Thrombin generation") OR "Thrombin generation assay") OR "Endogenous thrombin potential") OR "Thrombin generation test") OR "Calibrated automated thrombography") OR "Calibrated automated thrombin generation") OR "Calibrated automated thrombogram") OR (("Thrombin/analysis"[-Mesh] OR "Thrombin/diagnostic use"[Mesh] OR "Thrombin/metabolism"[Mesh])))) AND (((((((("bleeding risk") OR "bleeding tendency") OR Bleeding) OR Bleeds) OR "Bleeding complications") OR "Blood loss") OR hemorrhage) OR (("Hemorrhage/blood"[Mesh] OR "Hemorrhage/complications"[Mesh] OR "Hemorrhage/drug effects"[Mesh])))

Newcastle-Ottawa Scale for Assessing the Quality of Nonrandomized Sties in Meta-analyses

Newcastle-Ottawa Quality Assessment Scale—Case–Control Studies

Note: A study can be awarded a maximum of one star for each numbered item within the Selection and Exposure categories. A maximum of two stars can be given for Comparability.

Selection

1. Is the case definition adequate?
   a. Yes, with independent validation ★
   b. Yes, e.g., record linkage or based on self-reports
   c. No description.
2. Representativeness of the cases
   a. Consecutive or obviously representative series of cases ★
   b. Potential for selection biases or not stated
3. Selection of controls
   a. Community controls ★
   b. Hospital controls
   c. No description
4. Definition of controls
   a. No history of disease (endpoint) ★
   b. No description of source

Comparability

1. Comparability of cases and controls on the basis of the design or analysis
   a. Study controls for ___________ (Select the most important factor.) ★
   b. Study controls for any additional factor ★ (This criteria could be modified to indicate specific control for a second important factor.)

Exposure

1. Ascertainment of exposure
   a. Secure record (e.g., surgical records) ★
   b. Structured interview where blind to case/control status ★
   c. Interview not blinded to case/control status
   d. Written self-report or medical record only
   e. No description
2. Same method of ascertainment for cases and controls
   a. Yes ★
   b. No
3. Nonresponse rate
   a. Same rate for both groups ★
   b. Nonrespondents described
   c. Rate different and no designation

Newcastle-Ottawa Quality Assessment Scale—Cohort Studies

Note: A study can be awarded a maximum of one star for each numbered item within the Selection and Outcome categories. A maximum of two stars can be given for Comparability

Selection

1. Representativeness of the exposed cohort
   a. Truly representative of the average _____ (describe) in the community ★
   b. Somewhat representative of the average ___________ in the community ★
   c. Selected group of users, e.g., nurses, volunteers
   d. No description of the derivation of the cohort
2. Selection of the nonexposed cohort
   a. Drawn from the same community as the exposed cohort ★
   b. Drawn from a different source
   c. No description of the derivation of the nonexposed cohort

3. Ascertainment of exposure
   a. Secure record (e.g., surgical records) ★
   b. Structured interview ★
   c. Written self-report
   d. No description
4. Demonstration that outcome of interest was not present at the start of the study
   a. Yes ★
   b. No

Comparability

1. Comparability of cohorts on the basis of the design or analysis
   a. Study controls for _____________ (select the most important factor) ★
   b. Study controls for any additional factor ★ (This criteria could be modified to indicate specific control for a second important factor.)

Table 3 To be able to classify the quality of the studies, we defined the amount of stars as shown in the table

Stars (★)	(%)	Quality
1–3	<35%	Poor
4–6	35–70%	Moderate
7–9	70–100%	Good

Outcome

1. Assessment of outcome
   a. Independent blind assessment ★
   b. Record linkage ★
   c. Self-report
   d. No description
2. Was follow-up long enough for outcomes to occur
   a. Yes (select an adequate follow up period for outcome of interest)★
   b. No
3. Adequacy of follow-up of cohorts
   a. Complete follow-up—all subjects accounted for ★
   b. Subjects lost to follow-up unlikely to introduce bias—small number lost— >____ % (select an adequate %) follow-up, or description provided of those lost) ★
   c. Follow-up rate <____% (select an adequate %) and no description of those lost
   d. No statement
